# Quantitative proteomics reveals protein profiles underlying major transitions in aspen wood development

**DOI:** 10.1186/s12864-016-2458-z

**Published:** 2016-02-18

**Authors:** Ogonna Obudulu, Joakim Bygdell, Björn Sundberg, Thomas Moritz, Torgeir R. Hvidsten, Johan Trygg, Gunnar Wingsle

**Affiliations:** Department of Forest Genetics and Plant Physiology, Umeå Plant Science Centre, Swedish University of Agricultural Sciences, SE-90183 Umeå, Sweden; Department of Plant Physiology, Umeå Plant Science Centre, Umeå University, SE-90187 Umeå, Sweden; Department of Chemistry, Umeå University, SE-90187 Umeå, Sweden; Computational life science cluster (CLiC), Umeå University, Umeå, Sweden; Department of Chemistry, Biotechnology and Food Science, Norwegian University of Life Sciences, 1432 Ås, Norway

**Keywords:** Quantitative proteomics, Stepwise linear modelling, Aspen wood formation

## Abstract

**Background:**

Wood development is of outstanding interest both to basic research and industry due to the associated cellulose and lignin biomass production. Efforts to elucidate wood formation (which is essential for numerous aspects of both pure and applied plant science) have been made using transcriptomic analyses and/or low-resolution sampling. However, transcriptomic data do not correlate perfectly with levels of expressed proteins due to effects of post-translational modifications and variations in turnover rates. In addition, high-resolution analysis is needed to characterize key transitions. In order to identify protein profiles across the developmental region of wood formation, an in-depth and tissue specific sampling was performed.

**Results:**

We examined protein profiles, using an ultra-performance liquid chromatography/quadrupole time of flight mass spectrometry system, in high-resolution tangential sections spanning all wood development zones in *Populus tremula* from undifferentiated cambium to mature phloem and xylem, including cell expansion and cell death zones. In total, we analyzed 482 sections, 20–160 μm thick, from four 47-year-old trees growing wild in Sweden. We obtained high quality expression profiles for 3,082 proteins exhibiting consistency across the replicates, considering that the trees were growing in an uncontrolled environment. A combination of Principal Component Analysis (PCA), Orthogonal Projections to Latent Structures (OPLS) modeling and an enhanced stepwise linear modeling approach identified several major transitions in global protein expression profiles, pinpointing (for example) locations of the cambial division leading to phloem and xylem cells, and secondary cell wall formation zones. We also identified key proteins and associated pathways underlying these developmental landmarks. For example, many of the lignocellulosic related proteins were upregulated in the expansion to the early developmental xylem zone, and for laccases with a rapid decrease in early xylem zones. We observed upregulation of two forms of xylem cysteine protease (Potri.002G005700.1 and Potri.005G256000.2; Pt-XCP2.1) in early xylem and their downregulation in late maturing xylem. Our data also show that Pt-KOR1.3 (Potri.003G151700.2) exhibits an expression pattern that supports the hypothesis put forward in previous studies that this is a key xyloglucanase involved in cellulose biosynthesis in primary cell walls and reduction of cellulose crystallinity in secondary walls.

**Conclusion:**

Our novel multivariate approach highlights important processes and provides confirmatory insights into the molecular foundations of wood development.

**Electronic supplementary material:**

The online version of this article (doi:10.1186/s12864-016-2458-z) contains supplementary material, which is available to authorized users.

## Background

Wood development is of outstanding interest both to basic research and industry due to the associated cellulose and lignin biomass production. Detailed information about molecular events involved in wood formation is essential for both fundamental understanding of numerous biologically and ecologically important processes, and exploiting wood, which is an extremely valuable natural resource (Gion et al., 2005 [1]). For example, such knowledge can be used in screening and genetics approaches to modify wood quality in desired ways (Mishima et al., 2014 [2]). Wood formation is known to be initiated in the vascular cambium and to involve undifferentiated cambial cells developing into phloem and xylem cells through the processes of division, expansion, secondary wall formation, lignification, and finally (in xylem) programmed cell death (Hertzberg et al., 2001 [3]). Notably, we have examined patterns of protein localization, and identified candidate genes involved in wood formation and spatial distribution in stems of *Populus* trees (Tuskan et al., 2006 [4]; Kalluri et al., 2009 [5]; Nilsson et al., 2010 [6]). However, the amounts and composition of plant cell walls vary substantially amongst species, organs and cell types. The composition even varies substantially within cells’ walls. These variations ultimately dictate wood quality and abundance, but the regulatory mechanisms involved are far from fully understood. Thus, further elucidation is required of both the agents and processes that regulate cell identity, and cell wall synthesis, composition and abundance within wood formation zones (Zhong et al., 2007 [7]).

Such efforts may be greatly facilitated by detailed proteomic analysis, which is being increasingly used to explore mechanisms involved in the formation and differentiation of plant cells and organs (Gion et al., 2005 [1]; Kalluri et al., 2009 [5]; Nilsson et al., 2010 [6]). Mass spectrometry (MS)-based proteomic analysis is particularly valuable as it can provide data regarding not only protein expression, but also their structure (Albenne et al., 2009 [8]). Thus, proteomic studies have, for instance, provided information on numerous proteins of unknown function and identified various “housekeeping proteins” as important inducers or signaling elements of developmental pathways (Takáč et al., 2011 [9]). Clearly, therefore, comprehensive proteomic profiling at high spatial resolution could not only pinpoint changes in levels of key proteins during wood formation, but also provide important indications of the pathways and key regulatory switches involved. However, characterization of protein expression in plant stems is complicated by the heterogeneous mixtures of cell types and the dynamic range in protein abundance across developmental zones (Abraham et al., 2013 [10]; Qiu et al., 2013 [11]). Partly for this reason previous studies on wood development have generally focused on one or a few developmental gradients, relatively large sections, or tissue samples collected from a few positions in developmental sequences (Gion et al., 2005 [1]; Mishima et al., 2014 [2]; Hertzberg et al., 2001 [3]; Tuskan et al., 2006 [4]; Kalluri et al., 2009 [5]; Nilsson et al., 2010 [6]; Zhong et al., 2007 [7]).

In contrast, in the study presented here, protein expression patterns across all wood development zones were examined, at very high spatial resolution, in 47-year-old *Populus tremula* (aspen) trees. Aspen was chosen partly because it is an important species, ecologically and economically, and partly because abundant genetic resources are available for the genus (Mishima et al., 2014 [2]; Tuskan et al., 2006 [4]; Kalluri et al., 2009 [5]; Nilsson et al., 2010 [6]; Zhong et al., 2007 [7]). The latter facilitated interpretation of the data, which were processed using the following stepwise systems biology approach. We first examined global developmental gradient-related patterns in the spatial data series using Principal Component Analysis (PCA), and then applied sequential Orthogonal Projections to Latent Structures (OPLS) to model transitions in protein expression profiles between successive developmental zones. This modelling strategy resulted in a transparent overview of wood formation, identification of differentially expressed proteins in successive developmental stages of (i.e. paired transition effects) and the detection of connections along the developmental series that are intrinsically linked by information flows. Proteins identified as being significantly associated with transitions between zones aided subsequent interpretation of the affected biological pathways and dynamic changes. This approach for explicitly investigating the entire wood formation process in a species, using several multivariate statistical tools, has not to our knowledge been previously reported. The results show that high-resolution proteomics analysis can provide valuable complementary information for large-scale transcriptomic datasets, facilitate plant systems biology modeling efforts, and (specifically in this context) enhance understanding of cell wall biosynthesis and plants’ developmental pathways (Pandey and Mann, 2000 [12]). This should facilitate efforts to locate important developmental regulators, particularly where protein expression complement gene expression data.

## Methods

### Plant materials and sampling

Four mature, wild *Populus tremula* trees (47 years old, 15 m high) growing at a site in northern Sweden (62^o^ 21’N, 19° 47’E) were sampled for the analysis on 7 July, 2010, as follows. Cross-sections (2 × 10 cm) were cut from each tree’s stem at ca. 3 m above the top-soil level, flash-frozen in liquid nitrogen and stored at −80 °C. Tangential cryosections, 20 μm thick (20 μm × 2 mm × 20 mm, ≈0.5 mg, fresh weight), through the wood formation zones (from the phloem through cambium to the mature xylem) within a single annual ring were subsequently prepared, following previously described procedures (Uggla et al., 1996; 1998 [13, 14]). All samples were stored at −80 °C until further use.

### Characterization of wood sections

**Fig. 1:**
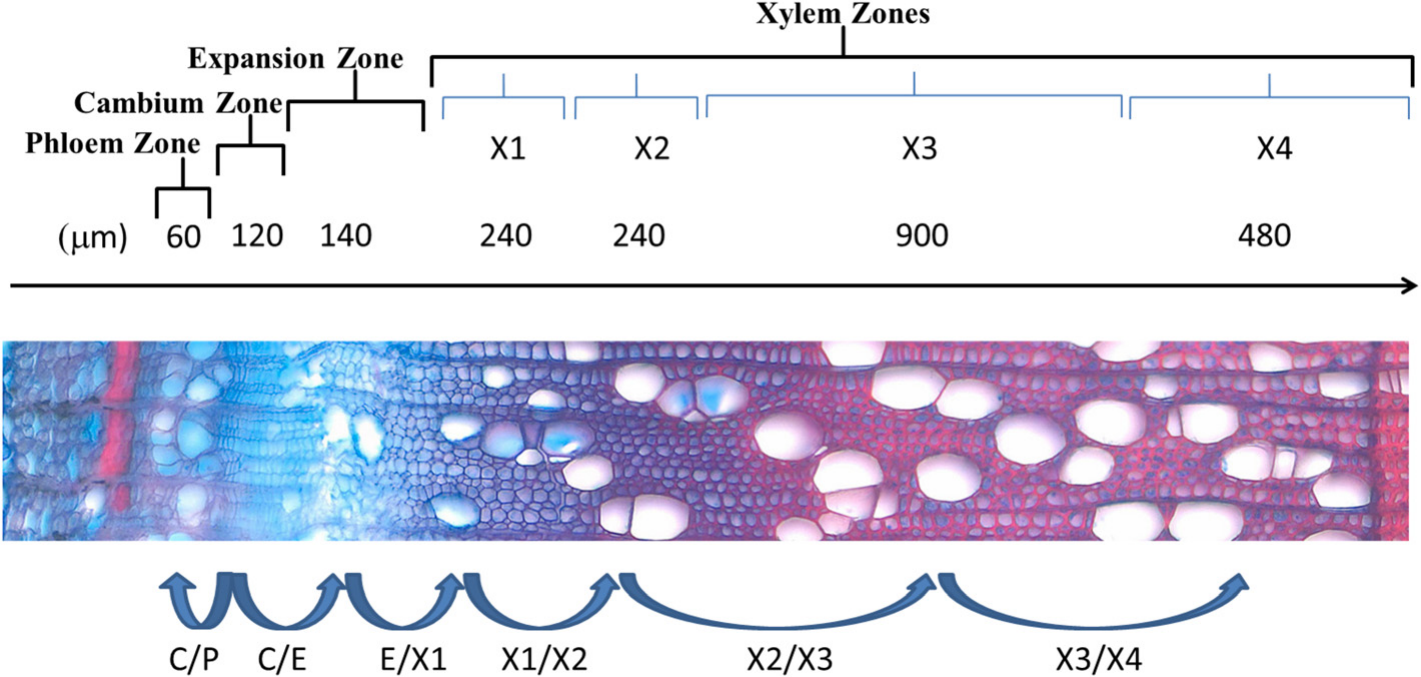
Schematic overview of transverse sections prepared from a specimen in tree 1. Illustration of the wood developmental zones defined as phloem, cambium, expansion zone and xylem, similar to illustrations in Hertzberg et al. (2001) [3] and Mahboubi et al. (2013). For details about the anatomical characterization and sampling scheme see (Additional file 1: Tables S1.1–4). Blue arrows indicate progressive trends across the series of development zones in the sampled wood (Symbols: C/P, cambium-phloem transition: C/E, cambium -expansion transition: E/X1, expansion-xylemX1 transition: X1/X2, xylemX1–X2 transition: X2/X3, xylemX2–X3 transition: X3/X4, xylemX3–X4 transition

The tangential cryosections were anatomically characterized as previously described by Uggla et al. 1996; 1998 [13, 14], in terms of wood development zones indicated in the safranin/alcian blue-stained section of stem from tree 1 shown in Fig. 1. In total, 482 tangential sections (110, 122, 126 and 124 from trees 1, 2, 3 and 4, respectively) were separately extracted, and some extracts were pooled prior to instrumental analysis following the sampling and pooling scheme presented in (Additional file 1: Tables S1.1–4). This resulted in 27 pooled samples for tree 1, and 28 for each of the other three trees. Based on the anatomical observations, some of the phloem and cambium zone sections were analyzed separately, while sets of three and nine pooled samples covered the xylem expansion zone and other parts of the xylem zone, respectively.

### Proteome analysis

Proteins were extracted from the cryosections largely following published procedures (Masuda et al., 2008 [15]). Briefly, 1 % sodium deoxycholate (SDC) in 10 mM DL-dithiothreitol and 50 mM ammonium bicarbonate (AmBic) solution was added to the 20 μm cryosections from each tree, mixed and heated to 95 °C for 15 min. The samples were then alkylated by adding 0.2 M iodoacetamide solution and incubating for 30 min at room temperature (RT) in the dark, then digested by adding 1 μg of trypsin (Promega/SDS Biosciences) in 50 mM AmBIc and incubating overnight (~16 h) at 37 °C. After adding trifluoroacetic acid (TFA) to a final concentration of 0.5 % w/v to stop the reaction and precipitate SDC, the samples were centrifuged at 14,000 *g* and the supernatants were cleaned using a C18 STAGE-tip (Pandey et al., 2000 [16]; Rappsilber et al., 2003 [17]). The resulting peptides were eluted with 0.1 % TFA in 65 % acetonitrile (ACN) and their concentration was measured using a Micro BCA Protein Assay Kit (Thermo Scientific, Cat. No. 23235). The eluates were then evaporated to dryness and stored at −80 °C until preparation for analysis by adding glycogen phosphorylase (50 fmol/μl) dissolved in 0.1 % TFA. Finally, their peptide contents were analyzed by reversed-phase liquid chromatography-electrospray ionization mass spectrometry (LC-ESI-MS), as follows.

First, the peptides were separated using a nanoACQUITY^TM^ ultra-performance liquid chromatography system (Waters, Massachusetts). 5 μl of each sample was loaded onto a PepMap100, nanoViper Acclaim® C18 trap column (100 μm i.d. × 2 cm, 5 μm particles, 100 Å pores; Thermo Scientific). The samples were then eluted from the trap column and separated on an HSS T3 (High Strength Silica T3) C18 analytical column (75 μm i.d. × 200 mm, 1.8 μm particles; Waters, Milford, MA), using a linear 80-min gradient of 1–40 % solvent B (3:1 ACN/2-propanol) balanced with 0.1 % aqueous formic acid (solvent A) at a flow rate of 300 nl min^−1^. The eluate was passed to a Waters SynaptTM G2 HDMS mass spectrometer equipped with a nanoflow ESI interface operating in positive ionization mode with a minimal resolution of 20,000. All data were collected in continuum mode and mass-corrected using Glu-fibrinopeptide B and Leucine Enkephalin as reference peptides.

### Protein identification and quantification

The data were processed with Protein Lynx Global Server v.3.0 (Waters) and the resulting spectra were searched against *Populus trichocarpa* v3.0 sequences compiled in the JGI Comparative Plant Genomics Portal database (http://phytozome.jgi.doe.gov) (Tuskan et al., 2006 [4]; Goodstein et al., 2012 [18]; Nordberg et al., 2014 [19]) along with sequences for human keratin and rabbit glycogen phosphorylase.

The database search settings were: enzyme-specific cleavage with one miss-cleavage allowed; oxidized methionine and protein N-terminal acetylation as variable modifications, peptide and fragment tolerance 10 and 25 ppm, respectively; and a false positive rate of 3 %. Detected proteins were quantified from the sum of the top three matched peptide intensities, as the average intensity of the three most intense peptides strongly correlates with absolute amounts of their source proteins (Silva et al., 2005 [20]; Distler et al., 2014 [21]). All samples were weighed prior to extraction and protein peak area data were normalized relative to volume of the tissue sample (total tissue normalization). Proteins were classified as found if at least one peptide was sequence-unique. In total, 13,017 unique peptides corresponding to 3,082 proteins were quantified and used in PCA of the samples from all trees, as implemented in the SIMCA version 14.0 software package (Umetrics, Umeå, Sweden). Differences amongst zones in the wood series were subsequently investigated in detail using OPLS and orthogonal partial least-squares discriminant analyses (OPLS-DA) models. Significance testing for differentially expressed proteins (the significance of changes in abundance of proteins, and their association with specific developmental stages/transitions) was done by calculating jack-knifing confidence intervals, setting α = 0.05 as the significance limit (Efron et al., 1983 [22]; Wiklund et al., 2008 [23]). More details are found in the Multivariate modelling paragraph.

Lists of all proteins and significantly differentially expressed proteins discussed in the paper can be found in (Additional file 2: Tables S2.1–8). The corresponding ID / Keyword of *Populus trichocarpa* gene models from Phytozome and *Arabidopsis thaliana* gene models from TAIR are indicated in the discussion when available, e.g. for Potri.009G067100.1 (Pt-TUB14; TUB6),

### Multivariate modelling

Modelling temporal and spatial variations in biological systems is essential for understanding their dynamic responses to external perturbations and/or endogenous developmental processes (Rantalainen et al., 2008 [24]; Lander, 2014 [25]). In the presented study we applied PCA, and OPLS regression to analyze spatial patterns in the data (which are also temporal patterns, as the spatial progression from cambial initials to mature phloem and xylem reflect chronological developmental sequences). Time and space cannot be disentangled and the location of a sample in a dynamic series is also time-dependent [26]. These procedures enabled visualization of the main developmental patterns and identification of proteins whose abundance monotonically increased or decreased along the developmental series (Rantalainen et al., 2008 [24]).

PCA is an unsupervised pattern recognition method that projects the main variation in a multivariate dataset into a low-dimensional subspace. OPLS divides the systematic variation in a matrix of descriptor variables (**X**) into two separate parts: a predictive part (denoted in the equations below by subscript $p$) that is correlated to a matrix of selected response variables **Y** and an orthogonal part (denoted in the equations below by subscript $o$) describing the variation that is not correlated to **Y** (Trygg & Wold, 2002 and 2003 [27, 28]). In the mathematical model for a single response variable or two-class discriminant analysis, the **X-**part of the OPLS can be written as:

$$ X=1\overline{x}\kern0.37em +\kern0.37em {t}_{p\ }\;{p}_p^{\hbox{'}} + {T}_{o\ }\;{P}_o^{\hbox{'}}+E $$

and the **Y** OPLS model prediction can be written as

$$ y=\overline{y} + \kern0.37em {t}_{p\ }\;{q}_p^{\hbox{'}}+f, $$

where *q*_*p*_^'^ and ***f*** are the loading and residual vectors, respectively, for y.

Estimation of multivariate sub-models between zones can be treated as a discriminant analysis problem, where each describes the transition between neighbouring zones. OPLS-DA can be used to describe these space (and time)-related protein expression patterns in the data. The resulting OPLS-DA predictive component effectively describes the change in the protein expression space. More specifically, the predictive loading vector ***p***_***p***_ describes the direction of change between the two zones and the corresponding score vector ***t***_***p***_ quantifies the magnitude of that change by its Euclidean norm.

**Fig. 2:**
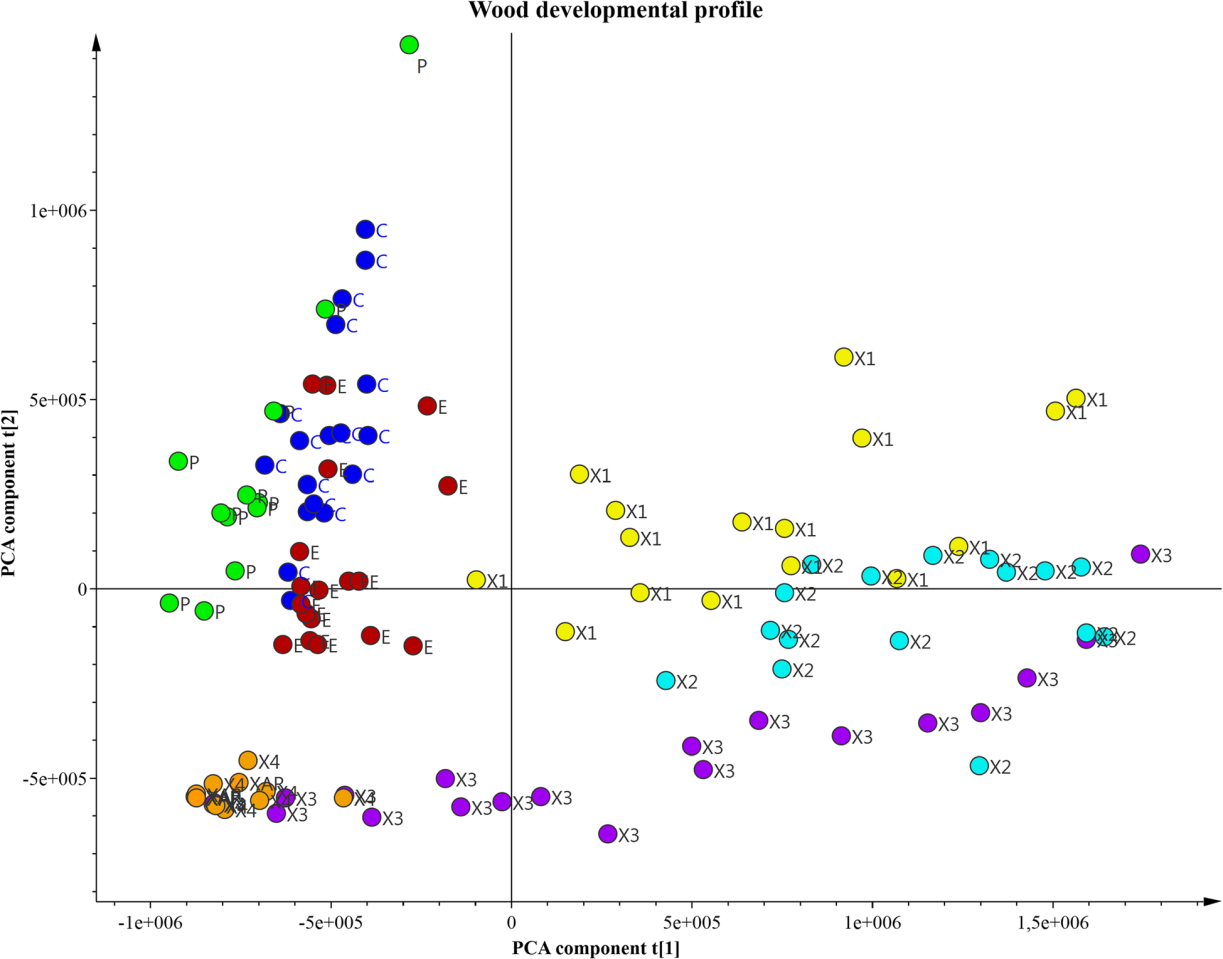
Joint genotype effect scores from the Global PCA model. The PCA model includes data from all samples, from all four sampled trees. The score plots revealed clear progressive trends across the series of development zones in the sampled wood. ID used in PCA score-plot; Phloem (P), cambium (C), expansion zone (E), xylem (X) (xylem divided into X1, X2, X3, X4 zones)

Prior to modelling, the datasets were column-centered with no scaling. First a PCA model based on all the samples and zones provided a global overview. Next OPLS-DA was used to model and identify the transitions between zones (that describes a change from one state to another), as previously described. In this analysis, the xylem zone was further sequentially classified into four segments (X1, X2, X3 and X4: from cambium inwards) to increase the resolution of the modelling. This division of the xylem zone was based on the PCA grouping (Fig. 2: Additional file 1: Tables S1.1–4). The OPLS-DA models collectively describe sequences of changes across the series of development zones in the sampled wood, from cambium outwards to mature phloem and from cambium inwards to xylem**.**

The stepwise transition method provides descriptive information and enables visualization of effects detected between paired transitions. This method examines and effectively describes linear changes in protein expression occurring during transitions between developmental stages across the wood series, as described in more detail in Rantalainen et al., 2008 [24]. An enhanced mathematical equation for the stepwise linear modeling can be written as:

and $$ d=\left|{\boldsymbol{t}}_{\boldsymbol{p}\ }\right| $$

$$ {\boldsymbol{p}}_{\boldsymbol{dist}}={\boldsymbol{p}}_{\boldsymbol{p}}\ d, $$

where ***p***_***dist***_ is defined as ***p***_***p***_, i.e. the direction of change, weighted by a scalar *d* defining the magnitude (size) of this change as determined from the local OPLS-DA model. The scalar *d* is calculated as the Euclidean norm of OPLS-DA model score vector ***t***_***p***_. Thus, for each stepwise OPLS-DA model, ***p***_***dist***_ represents the information about the direction as well as magnitude of change in the protein expression pattern between consecutive zones. A PCA model on all the resulting *p*_*dist*_ profiles provides an overview of the consecutive changes across the series of wood development stages.

Statistical parameters used to evaluate the multivariate models included R^2^, which defines the amount of variation explained by the model.

$$ {R}^2=1-SS(E)/SS(X), $$

where SS is the sum of squares, and X and E represent the column-centered data matrix and residual matrix, respectively.

The significance of the models was assessed using cross-validation (Wold, 1978 [29]; Wiklund et al., 2008 [23]).

Significance testing of proteins (the significance of changes in abundance of proteins, and their association with specific developmental stages/transitions) was done by calculating jack-knifing confidence intervals, setting *α* = 0.05 as the significance limit (Efron et al., 1983 [22]; Wiklund et al., 2008 [23]).

Original definitions, model statistics, selection criterion and detailed descriptions of the PCA, OPLS and its discriminant analysis variant are illustrated in Rantalainen et al., 2008 [24]; Trygg & Wold, 2002 and 2003 [27, 28].

### Pathway analysis

For robust biological interpretation of the transitions in protein profiles along the wood developmental series, pathways associated with the affected proteins were examined, using information obtained from the Kyoto Encyclopedia of Genes and Genomes (KEGG) database (Kanehisa and Goto, 2000; Kanehisa et al., 2012 [30, 31]) and MAPMAN (a user-driven tool providing pathway and biological process information; Thimm et al., 2004 [32]). Expression patterns and trends of the differentially expressed protein families across the series of wood development stages were visualized using PermutMatrix software v.1.9.3 (Caraux et al., 2005 [33]). These resources help assign putative functions to genes in the key affected molecular processes and have been shown to efficiently link proteins’ functions to biological pathways (Hucka et al., 2010 [34]; Srivastava et al., 2013 [35]).

All detected and differentially expressed proteins and corresponding identified pathways are listed in (Additional file 2: Tables S2.1–7).

Predicted subcellular localizations of the proteins were obtained from the Arabidopsis Information Resource (TAIR; http://arabidopsis.org) using ortholog information retrieved from the JGI Comparative Plant Genomics Portal database (http://phytozome.jgi.doe.gov) (Tuskan et al., 2006 [4]; Goodstein et al., 2012 [18]; Nordberg et al., 2014 [19]) and are listed in Additional file 2: Table S2.8.

## Results and Discussion

Wood formation involves sequences of cell division, differentiation and expansion that are initiated in the vascular cambium. Initiated at a specific lateral point in the cambium divide, cells differentiate into two files ultimately forming phloem composed of conducting sieve elements connected to companion cells and non-conducting parenchyma cells and fibers on the outer side, and xylem composed of conducting tracheary elements (TEs), and non-conducting parenchyma cells and xylem fibers, on the inner side (Carlsbecker et al., 2005 [36]). The vascular meristematic wood-forming tissues of aspen trees also include secondary xylem zones with defined boundaries. Thus, developmental stages across the wood-forming zone can be defined at high-resolution and explored to elucidate the underlying mechanisms, as shown in this study.

### Global and paired transition protein expression across the wood formation

In the first data analysis step, the PCA model of protein expression in all samples from all zones was examined (Additional file 1: Tables S1.1–4). The two-component PCA model explained 65 % of all variation in the full protein dataset (R2X = 0.65). The PCA score plot clearly shows that the data patterns are reproducible across the four replicate trees (Fig. 2), and revealed clear, progressive trends across the wood-forming zone. Phloem (P), cambium (C) and expansion zone (E) samples clustered in the top left quadrant, the X1, X2 and X3 xylem samples segregated mainly to the right, while xylemX4 samples clustered in the bottom left quadrant. There are also clear trends, not only between sequences of clusters, but also within clusters, as illustrated by the almost linear transition within and through the xylemX3 zone to the X4 zone.

**Fig. 3:**
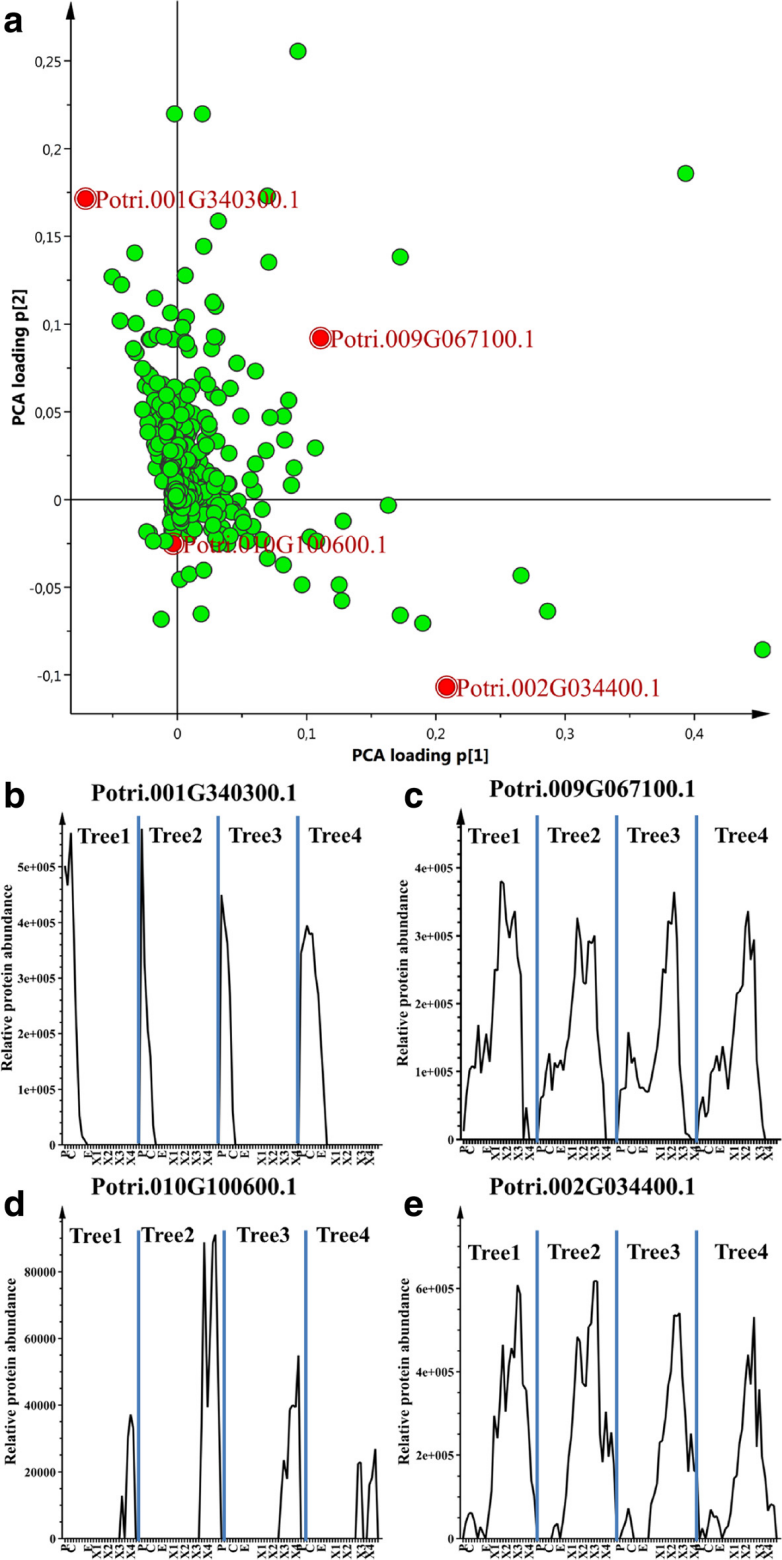
Raw data plots and loading plots from the Global PCA model. **a** Global PCA loading plots showing protein expression patterns across the series of development zones in the sampled wood. Raw data profiles of all samples, from all four trees for: (**b**) Potri.001G340300.1 (SEOR1) expressed in the phloem and cambium; Potri.009G067100.1 (TUB14), expressed from phloem to xylem zones; Potri.010G100600.1 (LTP) expressed in the xylemX3 and xylemX4 zones, and Potri.002G034400.1 (PCBER1) expressed in the xylemX2 and X3 zones as displayed in the four quadrants of the loading plot in A (for detailed information see Additional file 2: Tables S2.1–7)

The corresponding PCA loading plot (Fig. 3a) shows the importance of all the identified proteins for the first two Principle Components (PCs), including four whose expression profiles across the entire developmental sequence are shown in detail in Fig. 3b. The profiles of these proteins — Potri.001G340300.1 (Sieve-element-occlusion-related 1, EOR1), Potri.009G067100.1 (Tubulin, Pt-TUB14; TUB6), Potri.010G100600.1 (Lipid Transfer Protein, LTP) and Potri.002G034400.1 (Phenylcoumaran Benzylic Ether Reductase1, PCBER1) — in the phloem, cambium, expansion zone and xylem (X1-4) markedly differed, hence they were located in different quadrants of the loading plot in Fig. 3a (see also Additional file 2: Tables S2.1-7).

### Stepwise Phloem-Cambium-Expansion-Xylem transitions

Stepwise transition modelling involved the identification of proteins that were differentially expressed in successive stages of wood formation. A local OPLS-DA model was created for each transition between stages. All of these OPLS-DA models were significant according to cross-validation and CV-ANOVA (*p* < 0.05). The predictive component of each OPLS-DA model represents the size and change in protein expression in the corresponding transition.

A ***p***_***dist***_ profile was generated from each OPLS-DA model, representing the information about the direction and magnitude of change in the protein expression pattern between consecutive zones, as described above. These values are presented in (Additional file 2: Tables S2.2–7).

**Fig. 4:**
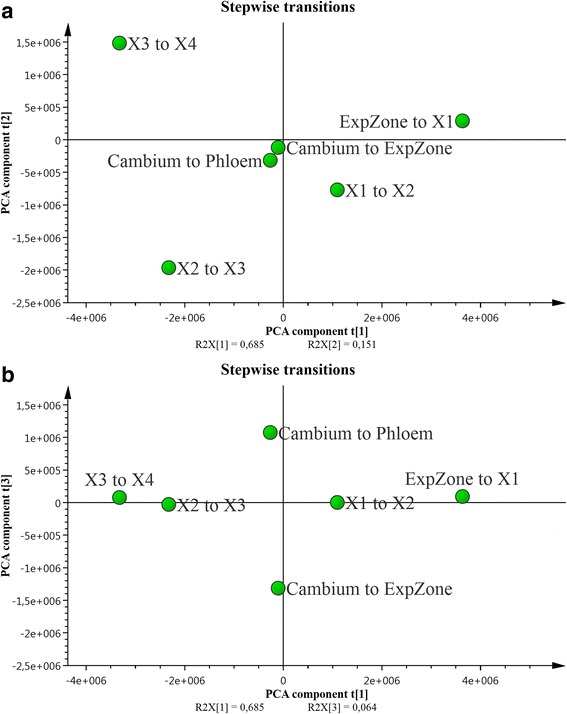
Joint genotype effect scores from the stepwise transition PCA models. PCA model overview of the consecutive development transitions in the sampled wood, i.e. Cambium to Phloem transition: Cambium to expansion zone (ExpZone) transition: E/X1, expansion zone (ExpZone) to xylemX1 transition: X1/X2, xylemX1 to X2 transition: X2/X3, xylemX2 to X3 transition: X3/X4, xylemX3 to X4 transition: (**a**) The t1–t2 PCA score plot. (**b**) The t1–t3 PCA score plot

A PCA model on all the resulting ***p***_***dist***_ profiles provides an overview of the consecutive changes in expression profiles across the series of wood development stages (C/P, cambium-phloem transition: C/E, cambium-expansion transition: E/X1, expansion-xylemX1 transition: X1/X2, xylemX1-X2 transition: X2/X3, xylemX2-X3 transition: X3/X4, xylemX3-X4 transition, Fig. 4). Scores for the first two components of the PCA model show very little separation of the cambium to expansion and cambium to phloem transitions. However, they substantially separate the expansion -xylem X1, X1-X2, X2-X3 and X3-X4 transitions, which lie in different quadrants of the t1-t2 score plot (R2X [1] = 0.68, R2X [2] = 0.15; Fig. 4a. While the first two components of the PCA model score plot (t1–t2) is dominated by the variation from the xylem related transitions, the third PC clearly separates the cambium-phloem and cambium-expansion transitions (R2X [3] = 0.06; Fig. 4b.

The t1–t2 score plot in Fig. 4a indicates that the first major change in the global protein expression pattern occurs in the expansion to xylemX1 transition, preceding deposition of the secondary cell wall. A weaker change in the same direction occurs along PC1, together with a shift in the opposite orientation along PC2, in the following xylemX1–X2 transition. This is followed by a strong and markedly different change in the xylemX2–X3 transition, which is located in the lower left quadrant of the t1–t2 score plot, almost opposite the expansion to X1 transition (Fig. 4a). This implies that most upregulated proteins become down-regulated (and vice versa) during the X2–X3 transition. The final xylem transition, X3–X4, is similar to the X2–X3 transition, in terms of PC1 component score, but changes influencing PC2 shift the X3–X4 position to the upper left quadrant.

The t1 versus t3 score plot (Fig. 4b) shows that changes in the cambium-phloem and cambium-expansion transitions are not only smaller in size or weaker (R2X [3] = 0.06) than changes in the other transitions (Fig. 4a), but also considerably different in nature, as they appear almost entirely in the third PCA component. Furthermore, they appear on opposite sides of PC3, indicating that they include changes in abundance of similar proteins, but the changes are largely in opposite directions. These observations strongly support the hypothesis that phloem- and xylem-forming programs are initiated at a dividing point in the cambium. Thus, the cambium-phloem and cambium-expansion transitions provide important complementary insights into wood development.

Moreover, the strong separation (in PCs 1 and 2) of the X3 to X4 transition is probably indicative of preparation for cell death. The downregulated proteins in the transition indicate that this process may include cessation of some amino acid conversions and carbon metabolism recycling (Additional file 2: Tables S2.5-7). Interesting indications of pathways that were up-and down-regulated in other zones can also be gleaned from looking at these tables. For example, in the cambium-expansion transition some signaling processes were upregulated, while in the expansion to xylemX1 transition, signaling, cell organization and phenolic secondary metabolism pathways were upregulated. Thus, the exploration patterns of global expression profiles across the series of development zones by PCA and sequential transitions by OPLS clearly identified informative stage-specific shifts in abundance of proteins, and pathways.

Previous studies have highlighted the roles of some proteins in specific zones, but analysis such as this provides opportunities for more holistic interpretation of the stage-specific profiles and processes, which is essential for understanding wood formation. Indications of the diverse biological functions of various groups of proteins that appear to participate in wood formation are discussed in more detail in the following sections.

### Carbohydrates, glycolysis and signaling

**Fig. 5:**
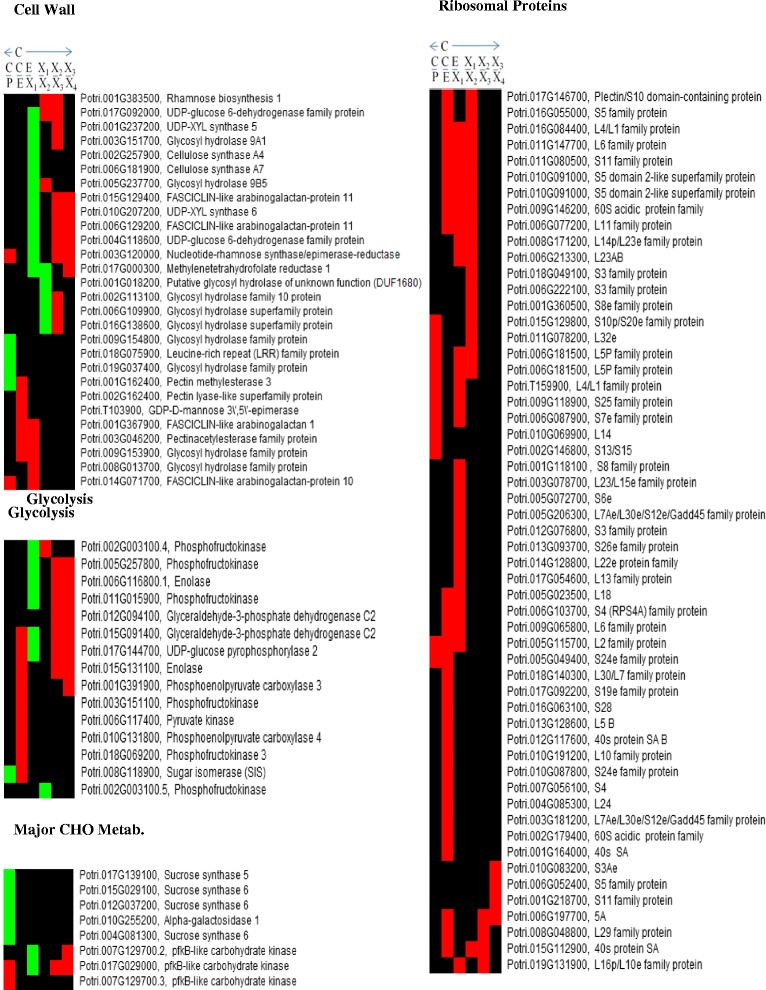
Visualization of patterns and trends of the differentially expressed protein families across the series of development zones from cambium outwards to phloem and inwards to xylem. Profiles of protein families encoding or involved in: ribosomal biogenesis, cell wall; glycolysis and major CHO metabolism. The red, green, and black color-codes show downregulation, upregulation and no change in expression, respectively, in indicated transitions. Symbols illustrated in Fig. 1: Symbols: C/P, cambium-phloem transition: C/E, cambium-expansion transition: E/X1, expansion-xylemX1 transition: X1/X2, xylemX1-X2 transition: X2/X3, xylemX2–X3 transition: X3/X4, xylemX3–X4 transition

Several sucrose synthase family proteins (Potri.015G029100.1; Potri.004G081300.2; Potri.017G139100.3 (SUS5); and Potri.012G037200.1) were significantly upregulated in phloem compared to cambium (Fig. 5, Additional file 2: Tables S2.2). Potri.002G202300.1 (Pt-SUS2.2; SUS3), another active sucrose synthase, was also detected in the phloem (Additional file 2: Tables S2.1). This analysis confirms these proteins to be in the phloem. Sucrose synthase family proteins have been classified as potential regulators of phloem functions (Schrader et al., 2004 [37]) and active sucrose transport from the phloem into the cambium region and beyond is important for wood formation (Mahboubi et al., 2013 [38]). A downregulation of fructokinase carbohydrate kinase family proteins (a splice form of Potri.007G129700.3 and Potri.017G029000.1) was observed in the cambium-phloem transition (Fig. 5, Additional file 2: Tables S2.2), with upregulation of Potri.017G029000.1 and a splice form of Potri.007G129700.2 in the expansion-xylemX1 zone. Potri.017G029000.1 was downregulated in xylemX2-X3 and xylemX3–X4 transitions (Additional file 2: Tables S2.2, 2.5, 2–6, 2.7) while Potri.007G129700.2 was downregulated only in xylemX3–X4 transition (Additional file 2: Tables S2.7). Microarray analysis of the genes Potri.017G029000 and Potri.007G129700 in hybrid aspen (*Populus tremula x P. tremuloides*) wood developing sections (Courtois‐Moreau et al., 2009 [39]) showed a similar expression in maturing xylem for the protein fructokinase carbohydrate kinases isoform Potri.017G029000.1 and the splice variant Potri.007G129700.2. Similar fructokinase expression has previously been reported by Roach et al. 2012 [40], presumably reflecting increased rates of fructose phosphorylation required to maintain carbon supplies and balance in developing wood. Some proteins involved in glycolysis were upregulated in the early xylem transitions and downregulated in the expansion and later-formed parts of the xylem transitions (X2–X3 and X3–X4) (Fig. 5, Additional file 2: Tables S2.1–7).

Xyloglucanases are important for primary cell wall expansion, and secondary cell wall remodeling. Several studies have suggested that their expression patterns strongly reflect their biological functions and their activity is reportedly concentrated in the middle lamella of mature fibers and developing secondary cell walls, possibly extending into mature xylem in *Arabidopsis *(Minic et al., 2007 [41]; Banasiak et al., 2014 [42]).

We also find an upregulation of Pt-KOR1.3 (Potri.003G151700.2) which has been hypothesized to be a xyloglucanase involved in cellulose biosynthesis in primary cell walls and reduction of cellulose crystallinity in secondary walls (Takahashi et al., 2009 [43]; Banasiak et al., 2014 [42]). We observed an upregulation of Pt-KOR1.3 in the expansion-xylemX1 transition and a downregulation in the xylemX2–X3 transition (Fig. 5), and hence Pt-KOR1.3 exhibits an expression pattern similar to that shown in previous studies.

Also splice variants of Pt-KOR1.3 (Potri.003G151700.3 and Potri.003G151700.4) and an isoform (Potri.001G078900; Pt-KOR1.2) were detected in our analysis but not found to be differentially expressed (Additional file 2: Tables S2.1). Analysis of the transcript Potri.003G151700 in hybrid aspen displayed a similar expression in maturing xylem for Pt-KOR1.3 (Courtois‐Moreau et al., 2009 [39]. Signaling G-proteins, Potri.006G057700.1 (RABA4D; RGP1), Potri.009G115000.2 (Pt-ACT2.1; RAB71) and Potri.013G123600.1 (Pt-RAB11.8; RABA1f) and mitochondrial electron transport/ATP synthesis F1-ATPase (Potri.009G137800.4, V-ATPase B subunit 2), which play important roles in systematic environmental adaptation and associated growth and developmental regulation in trees (Van Hemert et al., 2001 [44]; Zhao et al., 2005 [45]), were mainly downregulated in the cambium to phloem transition. Potri.009G137800.4 was also downregulated in xylemX2–X3 and xylemX3–X4 transitions (Additional file 2: Tables S2.7). The transcript expression showed a different pattern than the protein expression with an upregulation in the mature xylem for Potri.009G115000 and upregulation in the phloem and mature xylem for Potri.009G137800 (Courtois‐Moreau et al., 2009 [39]).

High energy-consuming processes, such as ribosome biogenesis and mRNA translation, could limit energy supplies and restrict translation capacity, thereby inhibiting cell growth and differentiation. Potentially reflecting mechanisms that counter this possibility, ribosomal proteins were highly downregulated at several examined wood development stages, particularly the cambium, expansion zone and late matured xylem (Fig. 5, Additional file 2: Tables S2.1–7). The strong downregulation of ribosomal protein biogenesis we observed in the xylem supports the hypothesis that it may participate in reprogramming the energy transformation and utilization machinery, inducing cells to switch to an energy preservation mode, in which only essential cell functions and viability are maintained when energy levels are low (Srivastava et al., 2013 [35]).

Thus, several carbohydrate related pathways, glycolysis and signaling processes involving agents such as sucrose synthase, fructokinase, G-signaling and ATPase appear to participate in regulation of the cambium to phloem transition.

### Cell wall and cellulose formation

**Fig. 6:**
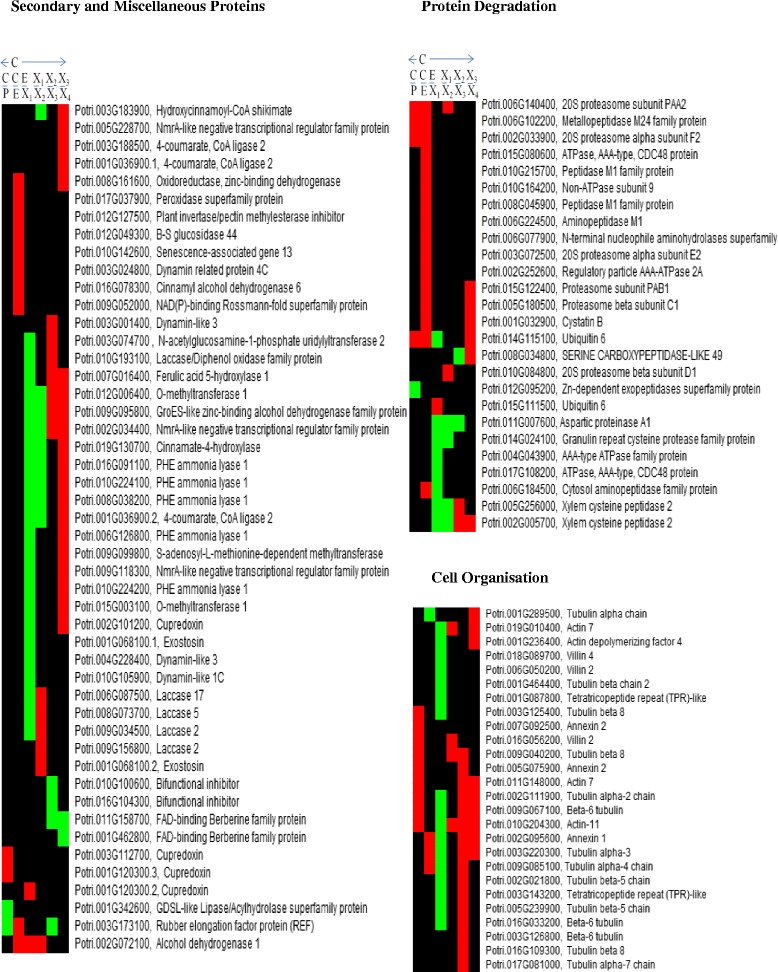
Visualization of patterns and trends of the differentially expressed protein families across the series of development zones from cambium outwards to phloem and inwards to xylem. Profiles of protein families encoding or involved in: cell organization, protein degradation and secondary metabolism and miscellaneous proteins. The red, green, and black color-codes show downregulation, upregulation and no change in expression, respectively, in indicated transitions. Symbols illustrated in Fig. 1: Symbols: C/P, cambium-phloem transition: C/E, cambium-expansion transition: E/X1, expansion-xylemX1 transition: X1/X2, xylemX1–X2 transition: X2/X3, xylemX2–X3 transition: X3/X4, xylemX3–X4 transition

Two fasciclin-like arabinogalactan proteins (Potri.001G367900.1, FLA1 and Potri.014G071700.2, Pt-FLA8.1; FLA10) were downregulated in the cambium-phloem and cambium-expansion transitions, while others FLA11 (Potri.006G129200.2 Pt-FLA11.1 and Potri.015G129400.1, Pt-FLA14.7) were upregulated in the expansion-xylemX1 transition then subsequently downregulated in the xylemX3-X4 transition (Fig. 6, Additional file 2: Tables S2.1–7). The Pt-FLA14.7 transcript showed a similar expression pattern as the protein in xylem (Courtois‐Moreau et al., 2009 [39])

The observed upregulation could reflect their role as adhesive molecules (Elkins et al., 1990 [46]) in fiber initiation/elongation and cellulose deposition, which increases stem strength (MacMillan et al., 2010 [47]). Although previous studies have suggested their involvement in many aspects of plant growth and development, their overexpression in cotton leads mainly to increases in fiber length and primary cell wall biosynthesis, specifically in the xylem region (Huang et al., 2013 [48]). Also the antisense expression of the fasciclin-like arabinogalactan protein FLA6 gene in *Populus trichocarpa* PtFLA6 gene have been shown to be specifically expressed in the xylem of mature stem, and the PtFLA6 protein distributed ubiquitous in plant cells with peak expression in stem xylem fibres (Wang et al., 2015 [49]). The downregulation in the latter stage of the xylem development indicates that they may have functions in processes other than cell wall biosynthesis, while the observed up- and down-regulation may reflect their participation in some rearrangement mechanism during cell expansion. Accordingly, *Arabidopsis* mutants with impairments in the Potri.015G129400.1 homolog At5g03170 reportedly have irregular or collapsed xylem (Turner et al., 1997 [50]; Persson et al., 2005 [51]). A co-expressed gene (At2g35700) also hypothetically acts as a regulator of secondary wall metabolism, inducing similar accumulation of suberin-like lipid polyesters in cell walls, but it is not typically involved in the deposition of lignin and cellulose, in contrast to other fasciclin-like arabinogalactan proteins (Lasserre et al., 2008 [52]).

A major component of primary cell wall in poplar is pectin, and changes in pectin methylesterases are useful early markers of cambial differentiation into either phloem or xylem (Guglielmino et al., 1997 [53]). Pectin methylesterases have been shown to act as negative regulators of symplastic and intrusive growth of developing wood cells in tissues of hybrid aspen, causing changes specifically in expanding wood cells (Siedlecka et al., 2008 [54]). Pectin acetylesterase protein Potri.003G046200.2 was downregulated in the cambium-expansion and expansion-xylemX1 transitions while the pectin methylesterase protein Potri.001G162400.1 was upregulated in the cambium-phloem transition and downregulated in the cambium-expansion transition. A similar expression pattern was found for the transcript of Potri.001G162400 (Courtois‐Moreau et al., 2009 [39]). These observations indicate that pectin is degraded and intrusive growth is regulated during formation of wood tissues.

Studies have focused on the protein levels of cellulose which is the main chemical polymer of wood and quantitative proteomics understanding of proteins directly involved in cellulose biosynthesis during wood formation in *Populus trichocarpa* (Loziuk et al., 2015 [55]). Cellulose and lignin are deposited in both primary and secondary cell walls, but mainly in the latter. Several cellulose synthase forms were identified, with Potri.002G257900.1 (CESA4) and Potri.006G181900.2 (Pt-CESA2.1; CESA7) upregulated in the transition from the expansion into xylemX1 zone, correlating with initiation of secondary cell wall formation (Fig. 5, Additional file 2: Tables S2.1–8). A similar expression pattern was found for the CESA4 transcript in the xylem (Courtois‐Moreau et al., 2009 [39]). Other cellulose synthases that were identified and play important roles in cell wall formation, but were not differentially expressed, include Potri.018G029400.1 (Pt-CESA1.2; CESA1), Potri.007G076500.5 (Pt-ATH.2; CESA6), Potri.011G069600.1 (CESA8) and Potri.005G194200.1 (Pt-CESA2.6; CESA9) (Additional file 2: Tables S2.1–8).

Lignin phenylpropanoid-derived polymers are mainly deposited in the secondary cell walls and this developmental feature differentiates secondary from primary cell walls. Lignin is essential for plant pathogen defense, mechanical support and rigidity. In addition, relative proportions of the monolignol polymers affect cell wall properties. Their composition, levels and arrangements may also differ among taxa, due to genetic variations, and among stages of wood development, thus clear elucidation of lignification is essential for understanding plant growth and development (Vanholme et al., 2010 [56]; Pesquet et al., 2013 [57]; Barros et al., 2015 [58]). The differentially expressed lignin-associated proteins included phenylalanine ammonia-lyases (PAL, Potri.006G126800; Pt-PAL1.2, Potri.008G038200; Pt-PAL.2, Potri.010G224100; Pt-PAL.3, Potri.010G224200, Potri.016G091100; Pt-PAL1.3), cinnamate 4-hydroxylase (C4H, Potri.019G130700, Pt-CYP73.3; C4H1); 4-coumarate:coa ligases (4CL, spliceforms of Pt-4CL.3 (Potri.001G036900.1; and Potri.001G036900.2) and Potri.003G188500; Pt-4CL.4, p-hydroxycinnamoyl-coa:quinate (HCT, Potri.003G183900), caffeoyl-coa o-methyltransferase (CCoAOMT, Potri.009G099800), ferulate 5-hydroxylase (F5H, Potri.007G016400; Pt-FAH1.4), caffeic acid o-methyltransferase (COMT, Potri.015G003100; Pt-OMT1.2), cinnamyl alcohol dehydrogenase (CAD, Potri.016G078300), peroxidase (PRX, Potri.017G037900) and laccases (LAC, Potri.006G087500, Potri.009G034500.1 Pt-LAC110c, Potri.009G156800, Potri.008G073700; Pt-GLAC90.1, Potri.010G193100) (Fig. 6, Additional file 2: Tables S2.1–8). Caffeoyl shikimate esterase (CSE), cinnamoyl-coa reductase (CCR) and ferric reductase-like transmembrane component (Rboh) family proteins were also identified, but not differentially expressed (Additional file 2: Table S2.1). The differentially expressed proteins were mainly upregulated in the transition from the expansion zone to the xylem zone indicating that active secondary cell wall and lignification biosynthesis occurred in the early xylem development. This was followed by downregulation in the X3–X4 transition, probably reflecting preparation for cell death (Figs. 5 and 6, Additional file 2: Tables S2.1–7). However it is likely that lignification occurs even though cell death has ended (Pesquet et al., 2013 [57]; Barros et al., 2015 [58]). Interestingly, laccases Potri.006G087500.1 (Pt-LAC110c; LAC17), Potri.008G073700.1 (Pt-GLAC90.1; LAC5), Potri.009G034500.1 (LAC2) and Potri.010G193100 showed similar increases in abundance from the expansion into xylemX1 zone, but an even more rapid decrease in the X1–X2 transition (in X2–X3 transition for Potri.010G193100) (Fig. 6, Additional file 2: Tables S2.1–7). The transcripts of Pt-PAL1.2, Pt-PAL.3, CCoAOMT (Potri.009G099800), Pt-OMT1.2, Pt-CYP73.3 showed a similar expression pattern as the proteins with an upregulation in xylem similar to that of Pt-LAC110c and Pt-GLAC90.1, but these genes showed a delayed decrease in mature xylem (Courtois‐Moreau et al., 2009 [39]). PRX (Potri.017G037900) transcripts also showed a downregulation, as the corresponding proteins, in the early xylem section. Pt-PAL.2 and CAD (Potri.016G078300) showed different expression compared to the protein; e.g. with the CAD transcript upregulated in the xylem and CAD (Potri.016G078300) protein expression downregulated in the cambium to expansion transition. These observations are similar to what was reported for expression of both cellulose and lignin biosynthesis genes in the xylem maturation phase in *Populus* (Courtois‐Moreau et al., 2009 [39]). Furthermore it supports the hypothesis of a postmortem lignification found in (TEs) in Zinnia elegans (Pesquet et al., 2013 [57]; Barros et al., 2015 [58]) to be present also in poplar, since the actual enzymes is downregulated while the lignification is still ongoing in mature xylem (Fig. 1, in X3 and X4 zones). Serine carboxypeptidase activity (like acyltransferase activity) has been linked to secondary cell wall formation and lignification (Porth et al., 2013 [59]). Serine carboxypeptidase-like 49 (Potri.008G034800.1, SCPL49) was upregulated in the xylemX2–X3 transition, then downregulated in the late xylemX3–X4 transition; a similar (but retarded) expression pattern compared to those of the lignin-associated genes (Fig. 6). Not all genes annotated as PAL or 4CL, for example, are presently identified in the literature as involved in lignin biosynthesis therefore there is the need for further experimental validation (Raes et al., 2003 [60]; Lu et al., 2013 [61]). However our results provide a valuable resource for tissue-specificity expression, investigation of isoform pattern and serve as a platform for further validation studies of proteins involved in lignification.

In summary, fasciclins, pectinases, cellulose synthases, serine carboxypeptidase and lignin-associated genes appear to play essential, tightly coordinated roles in cell wall and lignocellulose formation in aspen wood.

### Cell organization and protein degradation

Protein degradation is likely to be downregulated in the cambium-phloem and cambium-expansion transitions, because some proteases were downregulated in this zone, while others were upregulated in the expansion-xylemX1 transition (Fig. 6, Additional file 2: Tables S2.1–8). For example, we detected upregulation of forms of xylem cysteine proteases (Potri.002G005700.1, Potri.005G256000.2; Pt-XCP2.1) and granulin repeat cysteine protease (Potri.014G024100; Pt-RD21.1) in the early expansion-xylemX1 transition. The xylem cysteine proteases were downregulated in the later xylem transitions (X2–X4). Potri.002G005700.1 has not been previously reported in poplar, while Pt-XCP2.1 transcript was induced in the xylem in *Populus* stem (Courtois‐Moreau et al., 2009 [39]). The *Arabidopsis* cysteine protease homologue XCP2 reportedly participates in regulating autolysis of xylem TEs, which is essential for their programmed cell death (Avci et al., 2008 [62]).

We also observed marked expression of two families of cytoskeleton-related proteins, tubulins and villins, in the xylem. Tubulins play key roles in microtubule organization in cells, thus knowledge of their expression patterns is essential for understanding plant secondary cell wall development. Some tubulins are reportedly highly expressed in wood-forming tissues undergoing secondary cell wall thickening and their expression in xylem is associated with cellulose synthesis (Oakley et al., 2007 [63]). We observed that the tubulins Potri.002G111900.2 and Potri.009G085100.1 were both upregulated in the expansion- xylemX1 transition and downregulated in the xylemX2–X3 transition (Fig. 6, Additional file 2: Tables S2.1–8). In addition, Potri.002G111900.2 was downregulated in the cambium- phloem transition and Potri.009G085100.1 downregulated in the expansion-xylemX1 transition. A different expression pattern with an upregulation in mature xylem was found for the transcript of Potri.002G111900 (Courtois‐Moreau et al., 2009 [39]). Observations in developing cotton fibers have demonstrated strict temporal patterns in the expression of specific tubulin transcripts (Whittaker et al., 1999 [64]; Li et al., 2003 [65]) and Potri.002G111900 has previously been reported by McKown et al. 2014 [66] as a marker associated with biomass (log volume growth rate) traits in natural populations of *Populus trichocarpa*. Actin microfilaments are involved in plant cells’ vesicle delivery systems, shape determination, cytoskeleton formation and organization (Li et al., 2009 [67]). Isoform patterns were observed in the villin family, which are actin regulatory proteins. Our results showed that villin 2 family proteins in *Populus* contain some distinct isoforms, one Potri.016G056200.4 (Pt-VLN2.2; villin 2) with a downregulation in the cambium-phloem and xylemX1–X2 transitions and the other Potri.018G089700.1 (villin 4) an upregulation in the expansion-xylemX1 transition (Fig. 6, Additional file 2: Tables S2.1–8). A different expression pattern with an upregulation in mature xylem was found for the transcript of Potri.016G056200 (Courtois‐Moreau et al., 2009 [39]). Villin family proteins appear to participate in secondary cell wall formation and thickening.

## Conclusion

Our results confirm the utility of high-resolution mass spectrometric proteomic analysis for obtaining insights that complement transcriptomic data related to plants’ developmental processes. They also confirm the power of the approach based on PCA and OPLS regression for exploring the data series such analyses generate. The approach enabled clear visualization of the main developmental-related patterns of changes in expression profiles of global proteins, protein families and individual proteins. The first dramatic change in protein expression occurred in the expansion zone to xylemX1 transition. However, weaker but clearly crucial changes in protein expression (of opposite directions) occurred in the cambium-phloem and cambium-expansion zone transitions, supporting the hypothesis that a key division into phloem- and xylem-initiating cells occurs in the cambium.

In combination with pathway information, expression profiles of the differentially expressed proteins also enabled identification of upregulation and downregulation of several biological processes and pathways related to wood formation across the developmental sequence. Earlier studies of wood formation from specific developmental sections has been focused on gene expression (Hertzberg et al., 2001 [3]; Courtois‐Moreau et al., 2009 [39]). However, several investigations have only detected a weak correlation between transcript and protein expression, or failed to find a correlation altogether, and thus there is a need for proteomic analysis (Nie et al., 2007 [68]). Previous studies have highlighted roles of some proteins in specific zones, but the presented approach provides more comprehensive insights into wood formation processes. Moreover, the analysis identified the key proteins and associated pathways underlying these developmental landmarks. In summary, our data show that wood formation involves tight coordination of isoforms, splice variants and of multigenic protein families with distinct expression profiles; characteristic patterns of cytoskeleton formation, cell wall formation and organization; and integrated changes in activities of multiple biological processes and pathways.

## Additional files

Additional file 1:
**Table S1.1.** Anatomical characterization and pooling scheme for tree1. **Table S1.2.** Anatomical characterization and pooling scheme for tree2. **Table S1.3.** Anatomical characterization and pooling scheme for tree3. **Table S1.4.** Anatomical characterization and pooling scheme for tree4. (XLSX 35 kb) Additional file 2
**Table S2.1.** List of all identified proteins. **Table S2.2.** Proteins with significantly changing abundance in the transition from cambium to phloem. **Table S2.3.** Proteins with significantly changing abundance in the transition from cambium to expansion zone. **Table S2.4.** Proteins with significantly changing abundance in the transition from expansion zone to xylemX1. **Table S2.5.** Proteins with significantly changing abundance in the transition from xylemX1 to xylemX2. **Table S2.6.** Proteins with significantly changing abundance in the transition from xylemX2 to xylemX3. **Table S2.7.** Proteins with significantly changing abundance in the transition from xylemX3 to xylemX4. **Table S2.8**. Subcellular localizations of proteins in the wood series analysis sorted by location designation. (XLSX 348 kb)

## Abbreviations

PCA: Principal Component Analysis
OPLS: Orthogonal Projections to Latent Structures
SDC: sodium deoxycholate
AmBic: ammonium bicarbonate
TFA: trifluoroacetic acid
ACN: acetonitrile
LC-ESI-MS: reversed-phase liquid chromatography-electrospray ionization mass spectrometry
ESI: electrospray ionization
OPLS-DA: orthogonal partial least-squares discriminant analyses
KEGG: Kyoto Encyclopedia of Genes and Genomes
TEs: Tracheary elements
